# Selection bias in the reported performances of AD classification pipelines

**DOI:** 10.1016/j.nicl.2016.12.018

**Published:** 2016-12-24

**Authors:** Alex F. Mendelson, Maria A. Zuluaga, Marco Lorenzi, Brian F. Hutton, Sébastien Ourselin, for the Alzheimer's Disease Neuroimaging Initiative

**Affiliations:** a Translational Imaging Group, Centre for Medical Image Computing University College London, London, UK; b Institute of Nuclear Medicine, University College London, London, UK; c Centre for Medical Radiation Physics, University of Wollongong, NSW, Australia; d Dementia Research Centre, University College London, UK

**Keywords:** Alzheimer's disease, Classification, Cross validation, Selection bias, Overfitting, ADNI

## Abstract

The last decade has seen a great proliferation of supervised learning pipelines for individual diagnosis and prognosis in Alzheimer's disease. As more pipelines are developed and evaluated in the search for greater performance, only those results that are relatively impressive will be selected for publication. We present an empirical study to evaluate the potential for optimistic bias in classification performance results as a result of this selection. This is achieved using a novel, resampling-based experiment design that effectively simulates the optimisation of pipeline specifications by individuals or collectives of researchers using cross validation with limited data. Our findings indicate that bias can plausibly account for an appreciable fraction (often greater than half) of the apparent performance improvement associated with the pipeline optimisation, particularly in small samples. We discuss the consistency of our findings with patterns observed in the literature and consider strategies for bias reduction and mitigation.

## Highlights

• Demonstration and measurement of selection bias in AD classification experiments
• Bias accounts for much of the performance improvement seen with pipeline optimisation.
• Assessment of key risk factors and guidance on best research practices
• Evidence of selection bias in results collected by a recent literature review

## Graphical Abstract

**Figure:**
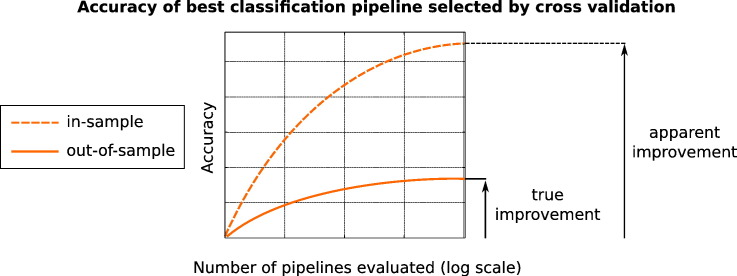

## 1 Introduction

Alzheimer's disease (AD) is probably the most studied application of supervised learning in neuroimaging (Arbabshirani et al., 2016, Klöppel et al., 2012, Orrù et al., 2012). Though AD progression is a continuous process, it is often approximated as a discrete one, making predictive modelling into a classification task. This may involve distinguishing AD subjects from healthy controls (HC), or, in a cohort of subjects suffering from mild cognitive impairment (MCI), distinguishing subjects who will progress to a full dementia diagnosis (MCIp) from those who will not (MCIs). This last task is of particular relevance, as the identification of subjects with expected rapid decline aids the detection of therapeutic effects in clinical trials (Klöppel et al., 2012).

A supervised learning pipeline comprises some combination of imaging, feature production, and supervised learning algorithm. After nearly a decade of research (Klöppel et al., 2008), hundreds of pipelines have already been developed and evaluated in the search for superior performance (Arbabshirani et al., 2016, Falahati et al., 2014, Mendelson). The field has been greatly assisted by large open access datasets provided by the Alzheimer's Disease Neuroimaging Initiative (ADNI), among whose key aims is the development of novel diagnostic methods (Weiner et al., 2013). Indeed, in recent years the vast majority of that research has been conducted using ADNI data (Arbabshirani et al., 2016, Falahati et al., 2014).

The use of supervised learning necessitates some form of cross validation (CV), broadly defined as the use of separate training and testing data (Arlot et al., 2010), to estimate predictive performance (Lemm et al., 2011). When individuals or collectives evaluate multiple pipelines using CV on a limited dataset in search of greater performance, it is inevitable that only relatively impressive results will be selected for publication. As a consequence of this, reported performance estimates will acquire a positive *selection bias* (Cawley and Talbot, 2010). Because AD is one of the most well studied applications of supervised learning in neuroimaging, it is also likely to be one of those where bias is most important. This bias should be of great concern to pipeline researchers, as it will mean that their results will fail to generalise. If a new pipeline is introduced into diagnostic practice on the basis of upwardly biased performance estimates and it actually fares worse than the system it replaces, then there is even the potential for harm.

Selection bias is not unknown in medical imaging (Rao et al., 2008), though the concept is often confused with the related phenomenon of overfitting. It is now common knowledge that when pipeline parameters are selected to maximise the full sample CV performance estimate, the ‘optimised’ performance estimate will acquire an optimistic bias (Arbabshirani et al., 2016, Kriegeskorte et al., 2009, Lemm et al., 2011). We want to draw attention to the fact that is not only *pipeline parameters* (e.g., the C of support vector machines) whose selection using CV can incur bias, but entire *pipeline specifications* (e.g., choice of features, algorithm); when the research community collectively evaluates many pipelines on the available data and selects the best results for publication, this is directly analogous to the selection of parameters so as to maximise performance. As such, the community should be aware of the potential of its published results to have acquired an optimistic bias.

This paper aims to assess the likely levels of selection bias in the existing results of the literature and to assess the relative importances of the key contributory factors to assist in the reduction and mitigation of bias in future. We consider the extent to which bias may be responsible for the improvements in classification performance observed with pipeline optimisation. Because selection bias is intimately related to the role of chance in determining the ranking of pipelines' CV performance estimates, we also consider the reliability of CV experiments in determining which of two pipelines is superior. To the best of our knowledge, no other study that has attempted to do this for either AD classification or any other application of supervised learning in neuroimaging.

Before we describe our experiment and its implications, we shall first provide a clear definition of selection bias and its relationship to overfitting.

## 2 Background

This section is concerned with providing a clear definition of selection bias and describing the mechanisms by which it may arise in AD classification research.

### 2.1 The performance of a pipeline

In this work, we are interested in the performances of *pipelines* themselves, rather than the performances of the fully specified prediction rules they produce. For the sake of simplicity, we define the performance of a pipeline to be the expected performance measurement obtained in the cross validation experiment being used, where the expectation is taken over many independent samples. This definitions allows us to separate concerns related to changes in training set size from those related to selection bias. For our purposes, in the absence of selection, CV performance estimators are by definition unbiased.

### 2.2 Introducing selection bias

The use of CV to select a high performing pipeline is directly analogous to the use of a single validation set to select a prediction rule (e.g., a regression curve) (Cawley and Talbot, 2010). In both cases, some noisy *in-sample* performance estimate based on a finite sample (e.g., CV accuracy measurement, in-sample mean squared error) is used to approximate the true *out-of-sample* performance on a larger population of items (e.g., true pipeline performance, expected mean squared error). There is some pool of candidate pipelines/rules, from which the one with the best in-sample performance estimate is selected. In rule selection, the size of this pool is often described by “model complexity” (Lemm et al., 2011).

The performance measurement for each candidate may be regarded as the sum of its true, out-of-sample performance plus a zero-mean random effect. Where random effects play a role in determining the empirical ranking of the pipelines by their in-sample performance estimates, the highest in-sample performance estimate is more likely to be associated with a positive random effect. (That is, the candidate with the highest in-sample performance is likely to have been ‘lucky’ in the validation experiment.) As a consequence of this, if the validation is repeated on an independent sample, the pipeline selected as the best in the first experiment will, on average, perform worse. The expected drop in performance is the *selection bias*. The term ‘selection bias' was first used in the context of prediction rule selection in Ambroise and McLachlan (2002). It was borrowed by Cawley and Talbot (2010) to describe the related phenomenon in pipeline selection. Selection bias may be considered a form of, or analogous to, the publication bias seen in group difference studies and clinical trials (Button et al., 2013, Easterbrook et al., 1991), as it describes the inflation of effects due to selective reporting.

**Fig. 1:**
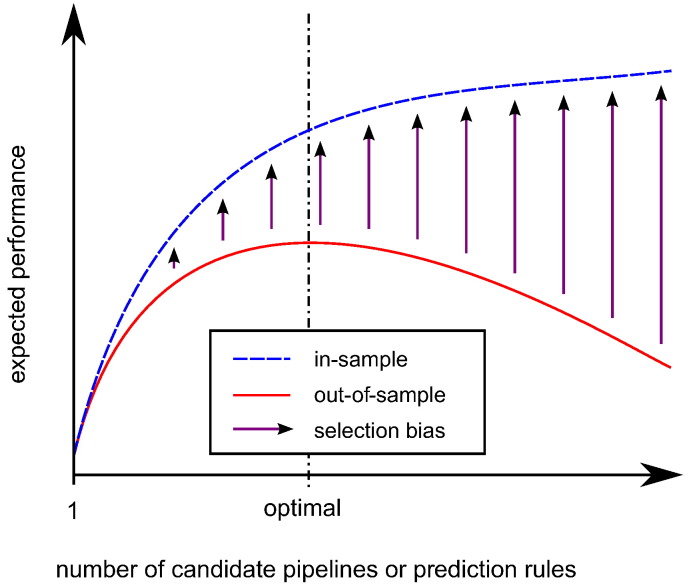
Illustration of expected in-sample and out-of-sample performances in pipeline or prediction rule selection. Selection bias is present as soon as there are multiple candidates. This may be contrasted with overfitting (i.e., deterioration in the out-of-sample performance), which only occurs once the number of candidates exceeds some optimal value.

Selection bias is related to, but distinct from, the related phenomenon of overfitting. Overfitting properly describes the situation where a pool of candidate pipelines/prediction rules considered for selection is expanded to provide more potential for improvement, but where this expansion actually leads to the selection of a candidate with an inferior out-of-sample performance than was the case before the expansion (Cawley and Talbot, 2010). This may be contrasted with selection bias, which is simply the expected difference between the in-sample and out-of-sample performances associated with a selected candidate. As illustrated in Fig. 1, while selection bias is necessary for overfitting, it also occurs in regular ‘fitting’. Fig. 1 may be viewed as an inversion of the more commonly seen illustration describing the relationship between error (or negative performance) and model complexity (related to candidate pool size), exemplified by Fig. 7 of Lemm et al. (2011).

### 2.3 Origins of selection bias in AD classification

Naked “double-dipping”, or the reporting of the in-sample performance estimate after it has been used to select continuous pipeline parameters (e.g., support vector machine regularisation), has long been recognised as unacceptable practice (Kriegeskorte et al., 2009, Lemm et al., 2011). However, it is not only pipeline's parameters whose selection may introduce bias, but whole *pipeline specifications* (e.g., choice of features, algorithm). While published works often proceed as if each new pipeline was fully specified in complete isolation from the data, this is often likely to be a fiction; in practice, because data are limited, research groups will inevitably reuse subjects as they evaluate sequential pipelines ideas. Years after proof of concept, new AD classification pipelines are only likely to be of widespread interest if they deliver improvement over state-of-the-art alternatives, and relatively unimpressive results are unlikely to appear in new publications. While some of the selection may occur through the rejection of manuscripts in review, the situation is likely to be more similar to that seen in clinical research, where the most common reason that unimpressive (non-significant) results go unpublished is that they are never even submitted for publication in the first instance (Easterbrook et al., 1991).

Selection can occur not only at the level of an individual research group, but at the level of an entire research field. The large collections of imaging data required for AD classification experiments are far too costly for each new study to reproduce. These are expensive and time consuming to produce, so it is not possible for each new study to use a new, independent dataset. Instead, studies typically draw data from large, open access repositories of the type provided by ADNI (Weiner et al., 2013). Indeed, in recent years, the vast majority of AD classification studies have used overlapping subsets of the ADNI dataset (Arbabshirani et al., 2016, Falahati et al., 2014). Where studies share data, they also share some of the random effects in CV performance estimation. This makes it possible for uncharacteristic performance results with large random effects, such as those produced by extensive selection, to be repeatable across studies. As the research community collectively evaluates new pipelines on the shared dataset to select the best for publication, it simulates the bias producing behaviour of the individual research group. This is the second challenge identified by Nowotny,^2^ who primarily considers classification in the MNIST dataset. It is recognised in the design of the Kaggle machine learning challenges,^3^ where it is dealt with by using a second validation dataset that is kept hidden until the end of the challenge.

## 3 Materials and methods

The organisation of the materials and methods is as follows: in Section 3.1, we describe the ADNI subjects and images we used; we then describe how we build our pipelines in Section 3.2 and the design of our experiments in 3.3, 3.4.

### 3.1 Subjects and imaging data

All imaging and clinical data were obtained from the ADNI database. A detailed description of them is provided in Appendix A. In this study, we use T1-weighted structural magnetic resonance images from the baseline time-point alone. This is the most commonly encountered imaging setting in AD classification research (Arbabshirani et al., 2016, Falahati et al., 2014), in part because it is one of the most easily achievable in clinical practice. Each image underwent the full pre-processing offered by ADNI for the relevant scanner type. This includes corrections for distortions due to gradient non-linearity (GradWarp) and corrections for additional image intensity non-uniformities (B1 correction and N3 histogram peak sharpening). Phantom-based spatial scaling was applied to all images. We conducted our own quality control assessment in addition to that provided by ADNI to cover subjects for whom no quality assessments were available. Where back-to-back images were available for the baseline time-point, the one with the superior quality score was selected.

The use of structural MRI alone allows us to have a larger total sample, something that is particularly important to us because our experiment design limits us to using less than half of the full number of subjects in a single CV experiment, and we wish to retain realistic sample sizes for that step. To further increase the size of our sample, we included images from both 1.5 and 3.0 Tesla scanners. This is not unprecedented (Abdulkadir et al., 2011, Ota et al., 2015), and it may be a sensible option for future diagnostic tools in clinical practice where the losses in performance due to heterogeneity may be outweighed by the gains due to increased training sample sizes (Abdulkadir et al., 2011). The inclusion of different field strengths may introduce small systematic differences between the representations of disease groups when they contain different proportions of images derived from each field strength, but we believe these should be negligible in our case (see Section 5.3).

Suitable baseline images were available for a total of 1437 subjects, and all of these underwent processing. For the purposes of this study, MCI subjects were considered stable if they had an assessment up to or beyond the subsequent 24 months follow-up period in which they were not given a diagnosis of AD. MCI subjects were considered progressive if they were given a diagnosis of AD at any point during the follow-up period. MCI subjects whose progression status could not be determined were excluded from the sample used for experiments. Subjects with suspected dementia aetiologies other than AD were excluded from our classification experiments. This yielded 372 HC subjects, 252 AD subjects, 230 stable MCI subjects (MCIs) and 135 progressive MCI subjects (MCIp).

#### 3.1.1 Further image processing

A sample specific group template was created using iterative affine and then B-spline registration using the publicly available NiftyReg package.^4^ Tissue segmentation and atlas propagation algorithms (more details in 3.2.2, 3.2.3 respectively) were applied to all images in their native space. Tissue segmentations and atlas labels were propagated to the space of the group template. Group templates parcellations were produced by combining the propagated labels of all 1437 available images by majority vote.

### 3.2 Pipelines

Due to the constraints of implementation and computational time, it would not have been possible for us to include many of the highest performing classification pipelines from the literature. Instead, we have tried to produce a large but plausible set of pipelines based on pairings of one of the 48 feature sets described in Section 3.2.1 and one of the 6 classification algorithms described in Section 3.2.6. Because one of the algorithms (random forest) cannot be combined with 24 of the (kernel-based) feature sets, there are 264 pipelines in total.

#### 3.2.1 Feature sets

We produce our 48 features sets in the same combinatorial way that we produce our pipelines. Each feature set is a combination of some imaging descriptor (see Section 3.2.2), one of two anatomical parcellation schemes to interpret that descriptor (Section 3.2.3), and some way of using that scheme to perform a knowledge-based feature selection (Section 3.2.4). All imaging descriptors were produced using no more than one of the two grey matter (GM) tissue concentration maps (see Section 3.2.2) and one of two anatomical parcellation schemes (see Section 3.2.3). Note that not all combinations of options were possible.

#### 3.2.2 Imaging descriptors

GM concentration maps are one of the most fundamental tools for the study of structural changes in the brain; they have a key role in the voxel-based morphometry that has become the established tool for group difference studies in structural neuroimaging. They were the first image descriptors considered for AD classification (Klöppel et al., 2008), and they are still studied frequently (Bron et al., 2015, Ota et al., 2015). Where we do not use GM concentrations to produce descriptors, we use the volumes of whole anatomical regions as descriptors themselves. Though these are used less often than GM measures, these also provide a straightforward description of brain atrophy that can be used to classify neurological diseases (Keihaninejad et al., 2012).

We can divide the imaging descriptors into two groups: *primal* descriptors representing the values of a quantity in each region of an anatomical parcellation in the native space, and dual or *kernel* descriptors represented by kernel matrices computed from voxelwise intensity scores in the groupwise space.

##### Primal descriptors

Regional GM loads were calculated by summing tissue concentrations over regions of the anatomical parcellations in the *native space* multiplied by the volume of the voxels. This produces descriptors that reflect changes in both the volume and concentration of GM. All GM loads were normalised for intra-cranial volume measured as the union of the white matter, GM and cerebro-spinal fluid tissue maps produced by SPM.

**SPM GM loads**. We use the publicly available SPM12 package^5^ to provide tissue concentrations maps.
**GIF GM loads**. The geodesic information flow (GIF) algorithm (Cardoso et al., 2012) used to produce the Neuromorph parcellations (see Section 3.2.3) jointly estimates various tissue concentrations maps. We included the resulting GM maps as an alternative to those of SPM.
**Anatomical region volumes**. The volumes of anatomical regions were normalised by intra-cranial volume.

##### Kernel descriptors

All kernel descriptors were produced from either GIF or SPM GM maps after transformation to the *groupwise space*. Kernel matrices were computed separately for each region of the group parcellation and then later combined by summation. There were three levels of further processing possible in the groupwise space, producing a total of six kernel descriptors.

1. **No further processing** as the simplest option.
2. **Modulation** by the Jacobian determinant of transform from the native to the groupwise space.
3. **Smoothing** (performed in addition to modulation) using an isotropic Gaussian kernel of 2.0 mm standard deviation (4.7 mm FWHM). This aims to compensate for registration errors and the spatial variation of atrophy patterns. The use of 2.0 mm was intended to be a middle-of-the road choice.

#### 3.2.3 Anatomical parcellation schemes

Due to its impact on the features produced, the choice of anatomical parcellation scheme can be an important determinant of a pipeline's performance (Ota et al., 2015). We include two parcellation schemes in our feature sets. These were chosen because they were the only ones for which we were able to obtain the sets of manually labelled images necessary for reliable atlas propagation (Cardoso et al., 2012).

**Hammers**. This is a scheme of 83 regions described in Gousias et al., 2008, Hammers et al., 2003, with 30 manually labelled reference images available online.^6^ The labels of these images were propagated to the space of our images and fused using the STEPS algorithm (Cardoso et al., 2013).
**Neuromorph**. This is a scheme of up to 141 brain regions provided by the commercial company Neuromorphometrics, Inc. under academic subscription. We use 35 labelled reference images originating from the OASIS project that were made available for the MICCAI 2012 Grand Challenge and Workshop on Multi-Atlas Labeling.^7^ These were propagated and fused using the GIF algorithm (Cardoso et al., 2012).

Each scheme offers a competing definition of what precisely constitutes an anatomical region in the brain. This in turn determines which areas of the brain will be used in predictive modelling. A notable difference between the two schemes is the way in which cortical regions are defined. In the Hammers scheme, many regions combine both the relevant cortical grey matter and the cerebral white matter beneath. By contrast, in the Neuromorph scheme, cerebral white matter and cortical grey matter are held in separate compartments.

#### 3.2.4 Spatial restriction/feature selection

The selection of relevant features can be used to remove variability in the data unrelated to the class discrimination problem. Knowledge-driven feature selection techniques have been shown to be superior to data-driven ones (Chu et al., 2012), and they have the added advantage of being computationally inexpensive. We consider two types of spatial restriction that implement a knowledge-driven feature selection.

**A symmetry constraint** enforced by combining, as an average, each pair of features related to a brain region occurring in each hemisphere. While the atrophy associated with AD may not be symmetric (Shi et al., 2009), the modes of atrophy that are most informative for classification may be. Due to the difficulty establishing a voxel-to-voxel correspondence between the hemispheres, the constraint was not applied to kernel-based feature sets.
**An exclusive focus on the temporal lobes** justified by their well established role in AD (Braak and Braak, 1991, Frisoni et al., 2010).

Zero, one, or both of these were applied to produce a feature set.

#### 3.2.5 Standardisation

All kernels were scaled so that the median inter-point distance in the whole sample was one. This scaling is mentioned because of its interaction with the C parameter in the SVM algorithm (see Section 3.2.6). All primal features were standardised by subtraction and division to ensure a zero mean and a unit variance.

#### 3.2.6 Classifier algorithms

Unless otherwise stated, all classifier algorithms were implemented in C++ by the authors.

**SVM**. The support vector machine (SVM) is the workhorse of neuroimage classification research (Kambeitz et al., 2015, Orrù et al., 2012), and is by a wide margin the most studied algorithm in applications related to AD (Falahati et al., 2014). We use the publicly available libsvm package (Chang and Lin, 2011) with a linear (precomputed) kernel, as is commonly preferred for the high dimensional classification problems of neuroimaging (Orrù et al., 2012). The C parameter was selected from the values 2^ −2^,2^ −1^,⋯ ,2^4^ using nested two-fold CV with five repeats. Results in preliminary experiments were essentially identical if the range of C values considered was expanded.
**RF**. The random forest (RF) classification algorithm is based on an ensemble of decision trees (Breiman, 2001). RF has been applied in AD classification (Falahati et al., 2014, Gray et al., 2013), as well as elsewhere in neuroimaging (Kambeitz et al., 2015). We follow the original specification by Breiman (2001) with the parameter mtry set to the rounded square root of the number of features. We used 100 trees. Increasing this number in preliminary experiments produced essentially identical results. As we know of no established way to apply RF to kernel features, combinations of the RF algorithm and kernel feature sets do not appear in our final set of pipelines.
**LDA1**. Linear discriminant analysis (LDA) classification has been used frequently in AD classification (Arbabshirani et al., 2016, Falahati et al., 2014), though less often than SVM. LDA is based on the projection of the data onto a single direction chosen to maximise the ratio of the between-class variance to the within-class variance. This variance is estimated using the assumption that the distributions of both classes have identical variance. For primal feature sets only, the Ledoit-Wolf lemma (Ledoit and Wolf, 2004) was used to provide a shrinkage estimator of the covariance. The threshold used for classification was based on a Gaussian model using class prior probabilities derived from the training set.
**LDA2**. This is an alternative version of LDA where the full sample covariance matrix is used, rather than the sum of the covariance contributions from each of the subject groups. This produces a biased, but slightly more stable estimator of the covariance. We have found this to fare better than LDA1 in synthetic high dimensional problems.
**NC**. The nearest centroid (NC) algorithm can be seen as something of a ‘control’ for the more complex linear methods (LDA, SVM), as comparison will show how important the estimation of the covariance structure is. This algorithm assigns unseen points the class whose distribution has the closest centre as estimated using the training data.
**KNN.** The K-nearest neighbours (KNN) algorithm assigns a class to each unseen subject based on the most common class among the K nearest subjects in the training data. Though it is very simple, KNN produces a flexible prediction model that can have high performance when the number of training examples is sufficiently high. Because the distances between points may be obtained straightforwardly from the kernel matrix, KNN may be applied to kernel-based problems. The number of neighbours was selected using nested two-fold CV with five repeats from the set of odd numbers less than one third of the size of the training set.

### 3.3 Cross validation and performance measurement

We consider repeated stratified K-fold CV as an estimator of pipeline performance. The numbers of subjects that appear in each fold are determined exactly as in the publicly available libsvm package (Chang and Lin, 2011). By *R×Kcv, we denote K-fold CV with R repeats*.

The performance measure from a single repetition of K-fold CV is assessed using the classification accuracy, defined as the fraction of subjects correctly classified. This is probably the most commonly reported performance metric for AD classification (Arbabshirani et al., 2016, Bron et al., 2015, Falahati et al., 2014).

### 3.4 Resampling experiment design

**Fig. 2:**
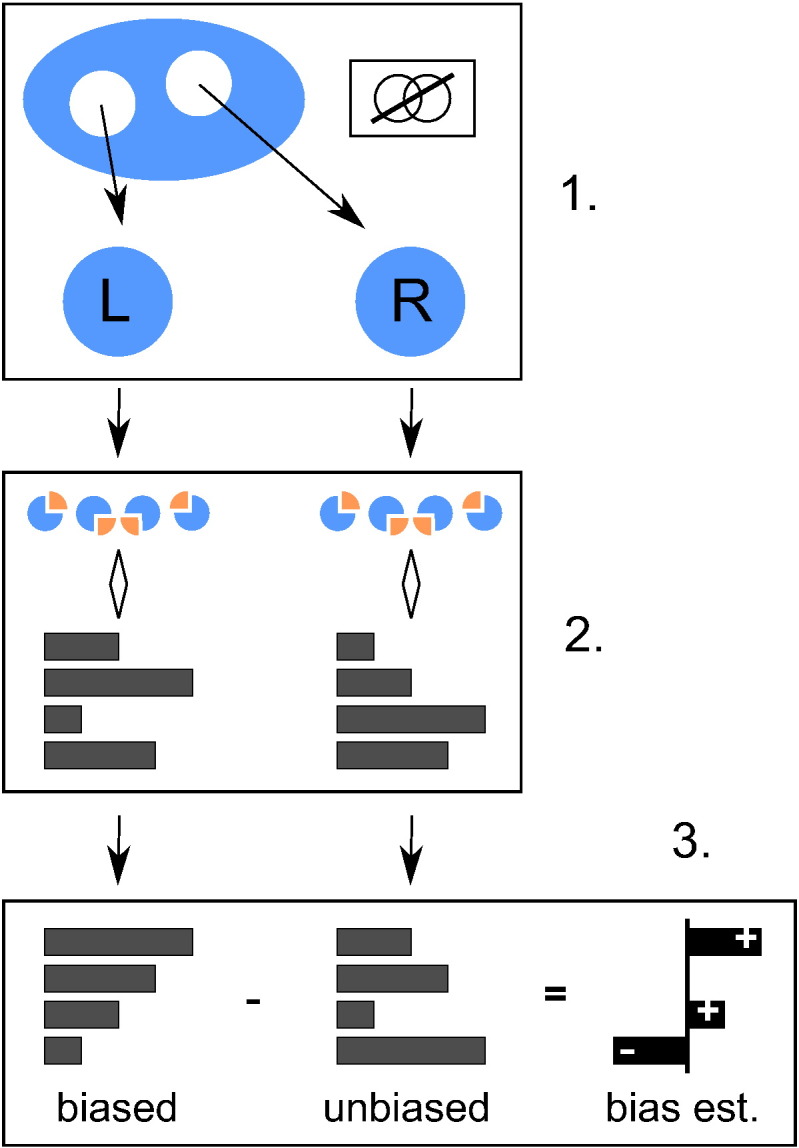
One iteration of the full experiment design described in Section 3.4. In step 1, two disjoint subsets labelled left and right are drawn from the full available dataset. In step 2, some form of CV is used to produce two independent performance estimates for a number of pipelines. In step 3, these are ranked by their values in the left subset. The difference between the two estimate sets is then taken as an estimator of the bias associated with the different rank positions.

In order to estimate the bias associated with selection of pipelines based on performance, one needs to obtain both biased and unbiased performance estimates. To do this, we use the resampling design described in Fig. 2 and defined below.

1. Two disjoint subsamples, respectively left and right, of a specified sample size and class composition are drawn randomly and without replacement from the full set of available samples.
2. Some form of (repeated) K-fold CV is applied to estimate the performance of the pipelines in the left and right subsamples separately. All pipelines are compared in parallel alongside one another.
3. The pipelines are ranked by their performance in the left dataset. The *n*th ranked performance measurement in the left dataset (henceforth, the in-sample estimate) is a biased estimator for the performance of the pipeline that obtained that rank (see Section 2.2), but the corresponding measurement in the right dataset (henceforth, the out-of-sample estimate) is not. The difference between the two is an (unbiased) estimator for the selection bias associated with the *n*th rank position.
4. The last step is repeated, but this time the roles of the left and right datasets are reversed. The average of the two resulting bias estimates is taken.

This process is repeated 2000 times using different random partitions into left and right subsets, and the bias estimates are averaged together to provide greater stability.

We use this design to investigate two classification tasks: the discrimination of subjects with AD from healthy controls (henceforth, AD detection), and the discrimination of MCI subjects who went on to progress to AD in a 24 month interval from those who did not (henceforth, MCI prognosis). Both tasks are conducted in samples containing only the two subject classes to be discriminated.

In order to investigate the effects of sample size and CV strategy, we repeat this experiment procedure many times while varying these parameters. When varying the sample size used for the left/right subsamples, we keep the ratio of positive and negative classes fixed at 2:3, a ratio which closely approximates that in the full available sample for both tasks (see Section 3.1). For AD detection, a subsample of size 50 will have 20 AD subjects and 30 controls. Similarly, for MCI prognosis, a subsample of size 50 will comprise 20 progressive subjects, and 30 who remained stable. Under this class balance constraint, sample sizes are varied from 30 up to the maximum permitted by the full available sample in steps of 10. We repeated all experiments using R ×Kcv strategies with *K* ∈{1,2,…,24} and *R* ∈{1,2,…,24/*K*}.

#### 3.4.1 Number of pipelines considered

We investigate the effect of the number of pipelines considered on selection bias. The probability of the pipeline obtaining the rank *p* out of *n* (with *p* = 1 denoting the lowest performance) being ranked the highest out of ***κ*** pipelines selected randomly without replacement is

(1) $$P_{\text{highest of}\kappa }=\left(\begin{array}{l} n-p\\ \kappa -1 \end{array}\right){\left(\begin{array}{l} n\\ \kappa \end{array}\right)}^{-1}.$$

By using this formula to combine the biases associated with all *n* ranks, we are able to measure the average in-sample and out-of-sample performances associated with the best performing pipeline over all $\left(\begin{array}{l} n\\ \kappa \end{array}\right)$ possible pipeline subsets.

We measure the progress associated with increasing the pool of candidate pipelines as the change in performance when moving from a subset of a smaller size t one of a larger size. The change in in-sample performance is the apparent progress anticipated based on a naive interpretation of results, while the change in the out-of-sample performance is the true progress expected on independent data.

#### 3.4.2 Decision power and representative variance

Selection bias is intimately related to a CV experiment's ability to correctly identify the best of several pipelines. We produce two summary measures to assess the ability of an experiment to do this.

The first measure is the *decision power*, defined as the posterior probability that an experiment correctly ranks a pair of pipelines. This is measured as the fraction of times that the in-sample performance ranking of a pair of pipelines was the same as the ‘true’ ranking based on the performance in all experiments, conditional on the absolute difference in in-sample performance estimates falling into a certain interval. This was averaged over all $\left(\begin{array}{l} 264\\ \;2 \end{array}\right)$ pairs. It was intended to help answer the question “What is the probability that pipeline A is actually better than pipeline B, given that pipeline A performed X% better in a CV experiment?”.

The second measure was the *representative standard deviation* of a pairwise difference in performance. For each pair of pipelines, the variance of the in-sample performance was computed. The average of all $\left(\begin{array}{l} 264\\ \;2 \end{array}\right)$ variances was then taken as a single representative variance for a difference in performance. The square root of this is taken as the representative standard deviation.

## 4 Results

Where the curves of multiple sample sizes appear in the plots of this section, these have been selected to display a representative range of behaviours. Where a single CV strategy is presented, this is 4×6cv. This was chosen for its relatively low bias and its intermediate fold number relative to the other strategies considered. Where both in-sample and out-of-sample accuracies are presented, the former is biased, while the latter can be regarded as accurate.

### 4.1 Accuracy of pipelines

We obtained a wide range of average pipeline performances in each combination of task, sample size, and CV strategy. We report the mean accuracies observed using 4×6cv and the maximum possible sample size for each task, as this produces the configuration most typical of the literature. For AD detection experiments on 300 subjects, the average performances of the pipelines spanned the range 70–90%. For MCI prognosis experiments on 300 subjects, the average performances spanned the range 60–70%. These accuracies are towards the lower end of the spectrum of published results (Arbabshirani et al., 2016, Cuingnet et al., 2011, Falahati et al., 2014, Weiner et al., 2013). A more detailed description of these mean performance results is presented in Appendix B.

### 4.2 Effect of rank on bias

**Fig. 3:**
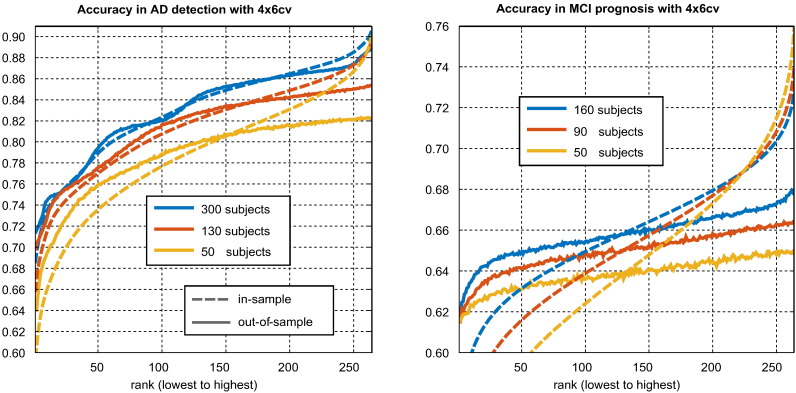
Illustration of the effect of ranking on performance estimation.

Fig. 3 presents the expected in-sample (apparent) and out-of-sample (true) performances associated with the different in-sample rank positions in 4×6cv. The selection bias associated with a rank position is the difference between its in-sample and out-of-sample performances.

As expected, both in-sample and out-of-sample performances increase with rank.^8^ The change in the in-sample performance is greater, as the lower and higher ranks are respectively associated with negative and positive selection biases. While higher performing pipelines are, on average, better than lower performing pipelines, the magnitude of the difference is exaggerated by the in-sample performance estimates. In AD detection, the exaggeration is relatively small, and the majority of the observed difference in in-sample performance is due to differences in the true, out-of-sample performance. In MCI prognosis, the majority of the difference is due to selection bias; for example, when using 160 subjects, the in-sample difference between the highest and lowest performing pipelines was on average roughly 16%, the out-of-sample difference between the two was on average only roughly 6%.

It can be seen that smaller samples increase the magnitude of the bias at all ranks. This effect is large enough to mean that the highest in-sample performance in MCI prognosis is higher when using 50 subjects than when using 160.

### 4.3 Variance as a determinant of bias

**Fig. 4:**
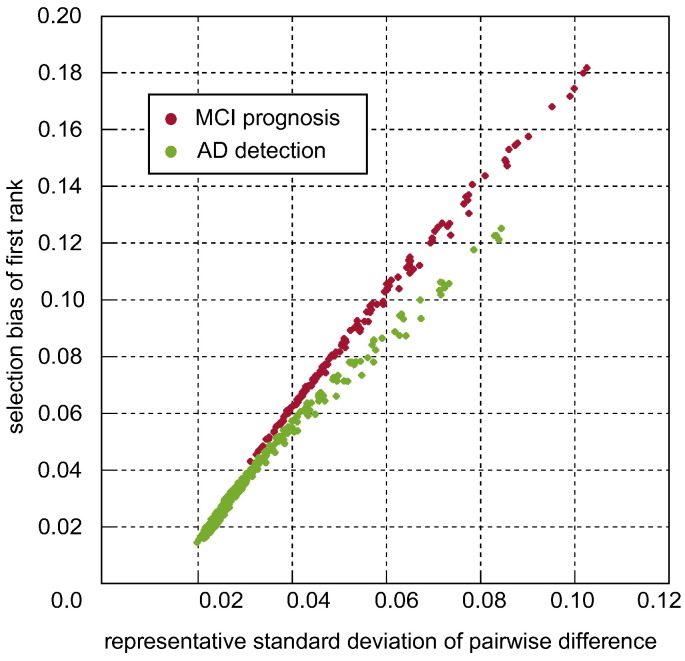
Illustration of the relationship between the representative standard deviation of a difference in performance and the selection bias associated with the highest ranking pipeline.

Fig. 4 plots the relationship between the representative standard deviation of a pairwise difference described in Section 3.4.2 and the bias associated with the highest ranking in-sample performance. Every sample size and experimental setting is represented by a point. The almost linear relationship demonstrates that variance in performance estimation is a key determinant of bias.

### 4.4 Bias as function of CV strategy and sample size

**Fig. 5:**
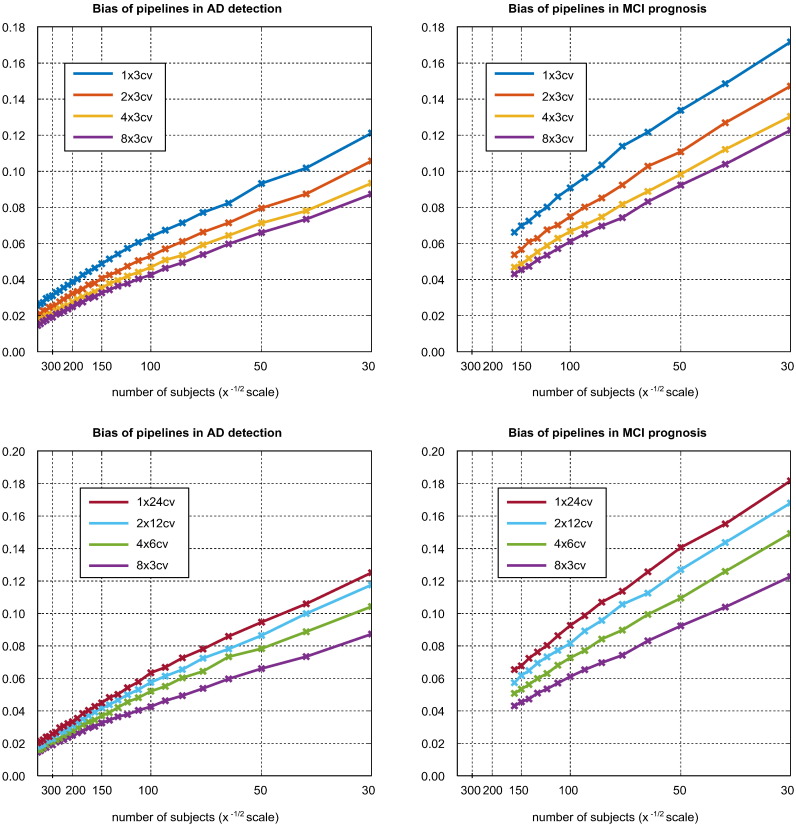
Effect of CV strategy and sample size on the selection bias associated with the highest ranking pipeline. The upper plots illustrate the effect of additional K-fold repetitions, while the lower plots compare strategies with approximately equal computational cost.

Fig. 5 illustrates the relationship between CV strategy, sample size, and the bias associated with the highest ranking pipeline. Through its effect on variance, sample size is an important determinant of bias, with bias being approximately proportional to its inverse square root. It can be seen that, for a given sample size and CV strategy, MCI prognosis is associated with greater bias than AD detection. In the upper plots, it can be seen that using additional K-fold CV repetitions decreases bias, though the returns diminish rapidly with the number of repetitions. The low value of *K* was selected because smaller values lead to more dramatic variance reduction with repetition. The lower plots compare CV strategies using equal train-test cycles, indicative of roughly equal computational effort. It can be seen that accepting a lower number of folds to produce a higher number of repetitions reduces bias appreciably.

**Fig. 6:**
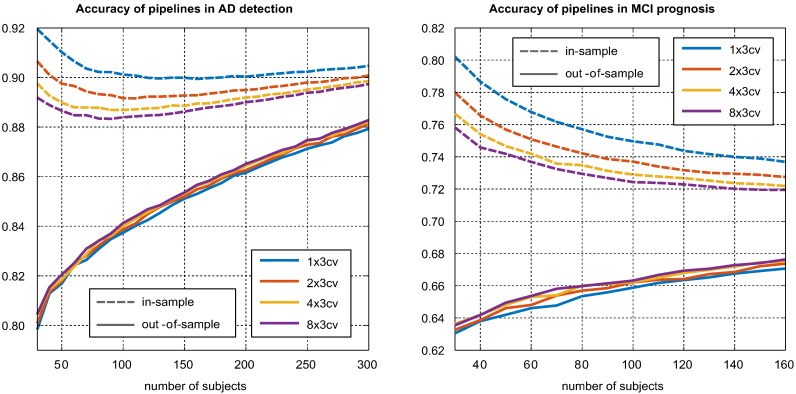
Effect of sample size on the in-sample and out-of-sample performances of the highest ranking pipeline. The association of low sample sizes and high selection bias introduces a paradoxical effect where smaller sample sizes can lead to greater apparent (in-sample) performance.

In Fig. 6, we present the relationship between sample size and the performance of the highest ranking pipeline. It can be seen that, through its effect on training set size and better pipeline selection, larger samples lead to higher true, out-of-sample performance. While this improvement will to some degree be reflected in the in-sample performance, there is also an unexpected effect; *because increasing sample size reduces selection bias (the difference between in-sample and out-of-sample performances), it can actually lead to a drop in the apparent (in-sample) performance of the highest ranking pipeline*. This effect only occurs at smaller sample sizes in AD detection. In MCI prognosis it is so large that it completely overwhelms the improvement expected due to the increase in the out-of-sample performance, and all the best in-sample performances are observed at low sample sizes. The use of a lower variance CV strategy (e.g., with more repetitions) decreases the in-sample performance estimate by reducing bias, but increases the out-of-sample performance by allowing for a better identification of a truly superior pipeline.

### 4.5 Real and apparent progress in pipeline optimisation

**Fig. 7:**
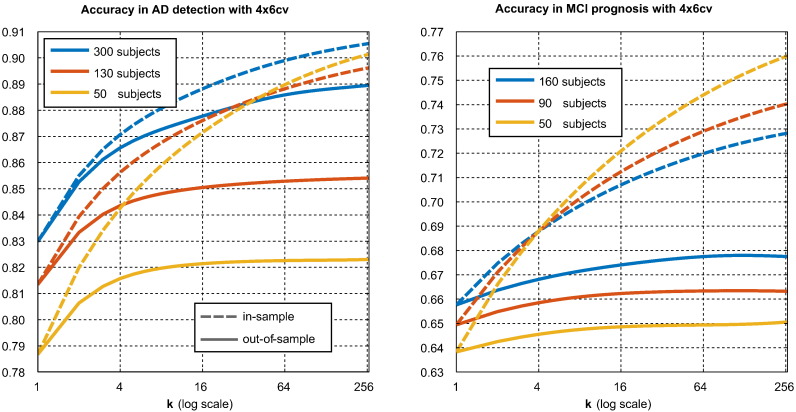
Effect of number of pipelines considered on the expected (biased) in-sample accuracy and (unbiased) out-of-sample accuracy associated with the highest ranking pipeline. The in-sample curve represents the apparent progress associated with pipeline optimisation, while the out-of-sample curve represents the true progress. It can be seen that much of apparent progress associated with an increasing number of pipeline options is spurious (i.e., due to increasing bias alone).

Fig. 7 illustrates the relationship between the number of pipelines considered, denoted ***κ***, and the in-sample and out-of-sample performances of the highest ranking pipeline. As described in Section 3.4.1, this is an average taken over all subsets of pipelines. The results of this figure may be compared with the theoretical model of Fig. 1.

When only one pipeline is considered, both in-sample and out-of-sample performances are the same, as there is no selection bias. As the number of pipelines considered increases, we can see the out-of-sample performance increase as the degree of opportunity for real improvement grows. The in-sample-performance of the best performing pipeline grows at a much faster rate, reflecting the faster growth of the selection bias. Both in-sample and out-of-sample performances increase sub-logarithmically (at a quickly diminishing rate). *Though there is significant selection bias, we observe no true overfitting.*

The change in performance seen when ***κ*** increases from 1 to some higher number represents the expected improvement in the performance of a selected pipeline seen when expanding the pool of candidate pipeline considered for selection from a single pipeline to the larger number. The change in the in-sample performance represents the apparent progress seen on the data used for selection, and the change in the out-of-sample reflects the true progress that would be repeatable on independent data. As can be seen in both halves of Fig. 7, while some of the apparent (in-sample) improvement in performance is due to real (out-of-sample) performance improvement, a large fraction of it is also due to increasing selection bias, particularly in MCI prognosis. Larger sample sizes are associated with lower bias and a larger fraction of true improvement.

**Fig. 8:**
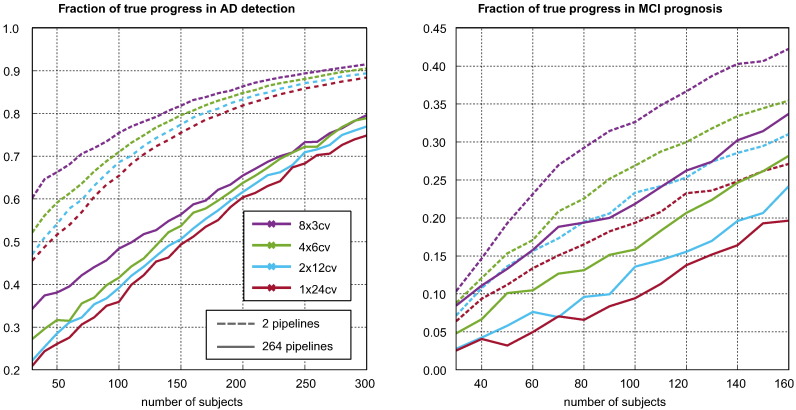
Illustration of the fraction of apparent (in-sample) performance improvement associated with pipeline optimisation that is due to real (out-of-sample) improvement in the performance of the selected pipeline. It can be seen that this fraction never rises to above 50*%* in MCI prognosis.

Fig. 8 considers the relationship between sample size, CV strategy and the fraction of the progress associated with pipeline optimisation that is repeatable. This is measured as the increase in the out-of-sample performance observed when expanding the pool of selectable pipelines divided by the increase in the in-sample performance. It can be seen that smaller sample sizes, higher variance cross validation strategies, and larger pools of candidate pipelines are associated with lower true progress fractions. *For MCI prognosis, the fraction of true progress never rises above 50*%*, indicating the majority of the improvement observed when using CV to identify superior pipelines is illusory.* For AD diagnosis, the majority of the apparent improvement is repeatable for sample sizes over 150.

### 4.6 Reliability of CV performance rankings

**Fig. 9:**
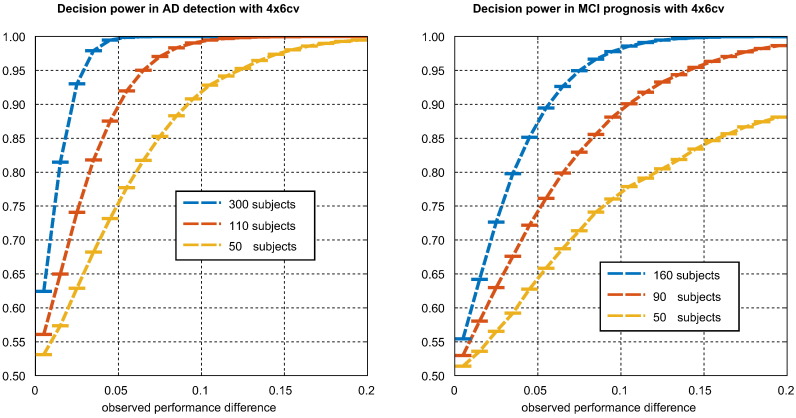
Illustration of the decision power associated with 4×6cv in both classification tasks. This is the probability that pipeline B is truly superior to pipeline A, given that B performed *X* better than A in CV experiment, where *X* is the value of the *x*-axis. Horizontal bars represent calculation intervals. For *i* ∈{1,2,…,20}, interval *i* contains difference *d* when (*i* − 1) < 100|*d*| ≤ *i*.

Fig. 9 illustrates the relationship between the magnitude of a pairwise difference in performance observed in a CV experiment, and the decision power, defined as posterior probability that the two pipelines associated have been correctly ranked. As expected, larger sample sizes dramatically increase the probability of a correct ranking. Decision power was computed for intervals of width 0.01 between 0 and 0.4. Too few differences in performance with magnitude above 0.4 were observed to estimate decision power accurately after that point.

**Fig. 10:**
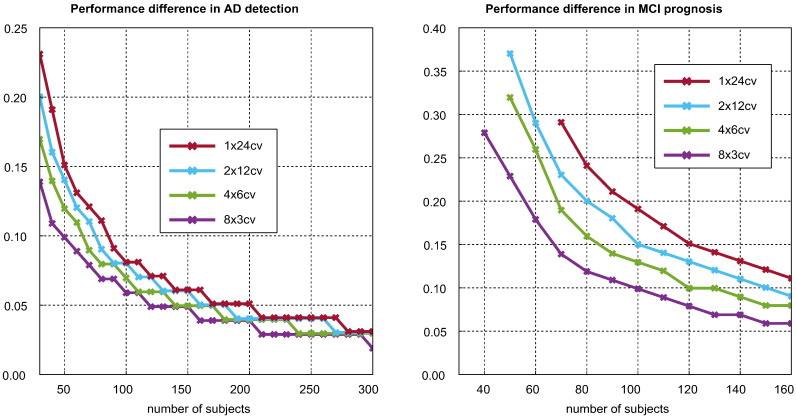
Illustration of the minimum observed difference in performance between two pipelines required to ensure a posterior probability that empirical ranking of the two is correct with at least 95*%* probability. Both *x*-axes begin at 30.

Fig. 10 considers the minimum magnitude required of an observed difference in performance to ensure a 95*%* posterior probability of a correct ranking. This was calculated as the lower end of the first interval to have a sufficiently high decision power. If one was not found, no point is plotted. It can be seen that low variance CV strategies and larger samples reduce the required difference. We found these minimum magnitudes required to produce a reliable ranking surprisingly large. If they are representative of the field, they highlight the importance of gathering a large sample before any pipelines are compared. We note that significantly larger observed performance differences are needed to reliably rank pipelines in MCI diagnosis than in AD detection.

### 4.7 Comparison with literature

The nature of selective reporting makes it difficult to assess the level of bias in the literature directly. One exception to this is found in challenges such as CADDementia (Bron et al., 2015), which report both biased in-sample performance estimates and unbiased out-of-sample performance estimates. As can be seen in Fig. 7 of the relevant paper (Bron et al., 2015), all 29 contestants overestimated the accuracy of their submitted AD classification predictors, with an average bias of almost 20*%* accuracy. Though it was not based on AD classification, a similar pattern is apparent in the MICCAI 2014 machine learning challenge,^9^ where all but two of the 48 submissions overestimated their performance. It should be noted that selection bias may be greater in challenges than in standalone studies, as researchers may be less concerned about reporting biased performance estimates where they know there will be an independent validation.

As seen in Fig. 6, selection bias can introduce a paradoxical effect where smaller sample sizes are associated with higher estimated performance. Larger sample sizes should allow for larger training sets, meaning that they should be associated with greater performance (Chu et al., 2012, Mendelson et al., 2014); if a negative association is observed, this may be taken as evidence of selection bias. This provides us with a way to look for selection bias in the AD classification literature. This is analogous to the use of funnel plots to detect publication bias in group difference studies (Sterne and Egger, 2001).

**Fig. 11:**
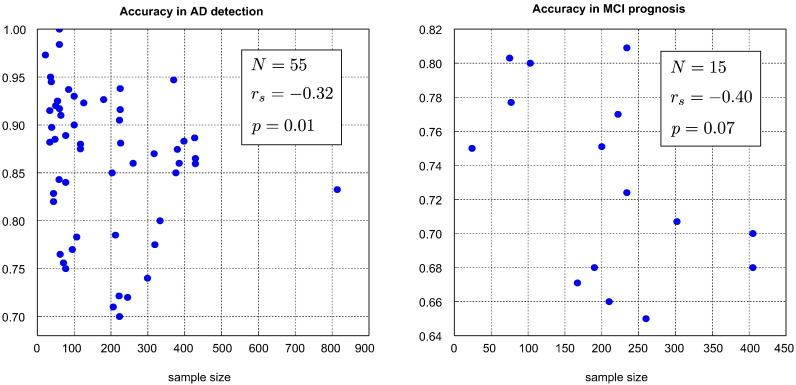
Summary of sample sizes and balanced accuracies from papers considered in Arbabshirani et al. (2016) using structural MRI alone. The selection of the studies and the production of performance measures is described in Section 4.7. The variable *r*_*s*_ denotes Spearman's rank correlation, and the *p* values are derived from a one-sided test against a non-negative rank association. *N* denotes the number of studies included in a tasks' plot.

To provide us with a representative set of reported AD classification results, we selected those from the recent review of Arbabshirani et al. (2016) (see Table 2 of that paper). We selected only those papers which advanced new methods (and thus excluded comparative studies such as Cuingnet et al. (2011)), and considered results for AD detection and MCI prognosis. In order to avoid performing additional selection ourselves, where a range of accuracies is reported, we took the mean of the upper and lower estimates. Multi-modal studies tend to be associated with smaller samples and higher performance. In order to avoid a spurious effect due to smaller samples being associated with more informative modalities, we included only those results achieved using structural MRI alone. This yielded 55 performance estimates for AD classification and 15 for MCI prognosis. These are presented in Fig. 11.

We conducted a one-sided test to assess the evidence against a non-negative association. Because a Gaussian null hypothesis is not appropriate, we used Spearman's correlation coefficient, rather than Pearson's. We observe a negative correlation in both AD detection and MCI prognosis. While the negative correlation is of greater magnitude in MCI prognosis, it only reaches significance in AD detection, due to the larger number of studies. We note that our test is somewhat conservative, as the null hypothesis of no selection bias actually corresponds to a *positive* rank association, rather than the zero rank association used in significance testing. We conclude that there is tentative evidence of selection bias in the AD classification literature.

## 5 Discussion

We have provided the first empirical demonstration of selection bias in AD classification. While we saw no true overfitting, our results demonstrate that bias can account for significant fraction of the apparent progress associated with pipeline optimisation (see Fig. 8). In small samples and MCI prognosis experiments, the fraction of progress attributable to bias was normally greater than 50*%*. The potential existence of such great bias should be of significant concern to researchers, as pipelines brought into clinical practice on the basis of overly optimistic results may lead to poor outcomes.

### 5.1 Controlling selection bias and mitigating its effects

Selection bias is principally controlled by variance in performance estimation and the number of pipelines considered for selection (see Fig. 4, Fig. 7). Selection bias may be reduced by using low variance CV strategies. We recommend that a large number of K-fold CV repetitions be used where computationally feasible. We recommend against the use of high variance strategies such as leave-one-out cross validation (LOOCV) (which is still used (Challis et al., 2015, Coupé et al., 2012, Dai et al., 2012, Dukart et al., 2013, Padilla et al., 2012)). Instead of using high numbers of folds, one can use a lower number of folds with a higher number of K-fold CV repetitions to produce a lower variance, lower bias CV strategy at equivalent computational cost (see Fig. 5).

Maximising sample size is also important for the avoidance of selection bias; selection bias is roughly proportional to the inverse square of the sample size (see Fig. 5). This is likely due to its effect on variance. We caution against any optimisation or comparison of pipeline specifications in small samples, as this is unlikely to produce reliable identification of superior pipelines (see Fig. 9, Fig. 10), and most of the apparent progress will be due to bias alone (see Fig. 8).

Though it would be cumbersome to implement, a selection bias free validation strategy does exist: one can treat the entire specification of a pipeline the same way one treats a continuous pipeline parameter, and select it using nested cross validation (Cawley and Talbot, 2010). While this provides an unbiased answer to the question “how well can we predict?”, it may not provide an answer to the question “what is the best way to predict?”. Outside of this, another valuable source of bias free results comes from classification challenges such as the recent CADDementia (Bron et al., 2015). Not only do challenges provide unbiased performance estimates for individual pipelines, but they can also offer an unfiltered overview of the full distribution of the performances of evaluated methods that may be contrasted with the literature. We note that if the best result in a challenge is selected, this reintroduces the potential for bias (Nowotny).

It is not only important that future studies work to minimise bias; the potential for bias must also be considered in the interpretation of previous published results. This is particularly the case where a result is linked with one of the following risk factors:

• the classification task is difficult (consider MCI prognosis versus AD detection);
• the sample used to demonstrate CV performance results is small; or
• there is evidence indicative of extensive selection; for instance, there are many adjustable settings in a pipeline, and there is not clear explanation for how they have been chosen.

That MCI prognosis is particularly susceptible to bias is troubling, as AD classification pipelines are primarily justified for the superior performance they are ultimately supposed to offer in that task. There are a number of other consequences of these considerations. For instance, practitioners may prefer simpler pipelines with fewer adjustable parameters over more complex alternatives, even when the latter come with higher reported performance. Furthermore, while it might be assumed that the apparent advantage of pipelines using alternative modalities instead of or in addition to structural MRI is due to additional information in the features, it is possible that some of it may be explained by the greater potential for selection bias afforded by the smaller samples available for validation.

### 5.2 Particularly susceptibility of AD classification to selection bias

AD classification is not unique among other applications of supervised learning to neuroimaging in being liable to bias, but there are good reasons why it may be particularly susceptible. The great societal cost of AD provides a strong incentive for pipeline optimisation, and the availability of open access datasets has made it considerably easier for researchers to contribute. As a consequence of this, the number of published pipeline specification is already in the hundreds (Mendelson), making it comparable to or greater than the size of a typical sample (Arbabshirani et al., 2016). While a larger number of pipelines specifications (combinations of pre-processing, feature selection, classification algorithm) considered for selection creates more potential for genuine performance improvement, it also creates more potential for bias.

### 5.3 Limitations

We do not provide a perfect simulation of the AD classification research field, as this would be technically infeasible; as such, our results should be interpreted as indicative rather than exact. Due to the difficulty in accurately determining and then implementing the full set of published research methods (registration, segmentation, feature selection, etc.) we have instead opted for a more limited set of relatively simple methods that nonetheless loosely representative of the field. Despite this, we still believe that our results are representative, and we do not anticipate that small changes to the precise combination of pipelines and CV strategies used would produce qualitatively different results. We note that while the number of pipelines considered for selection was large, it does not exceed the number of those already published (Mendelson). While the ranges of sample sizes considered do not include some of the largest numbers seen in recent studies, they still include those seen in the majority of AD classification studies (Arbabshirani et al., 2016).

We note that most of the metrics provided here (e.g., selection bias in a particular experimental setting) do not come with measures of measurement uncertainty. To produce these would require a deep statistical model of the distribution of results produced in our experiment, including a generative model for the distribution of subject representations in features space. We note that the same is currently lacking for even straightforward K-fold cross validation (Bengio and Grandvalet, 2004). This should not be a great concern, however, as the deviations due to randomness in our sample are likely to be smaller than those associated with changes in precise definitions of pipelines and experimental settings.

Variations in the field strength used to produce a T1-weighted image can have a subtle effect on the tissue concentration maps and region volumes derived from it. In comparative studies where subject groups contain different proportions of images from 1.5 and 3.0 Tesla scans, this may introduce spurious differences that are unrelated to physiology. Though this effect is likely present in our study, we do not think it is significant. The fraction of images acquired at 3.0 Tesla was relatively constant (between 31 and 48%) across disease groups (i.e., HC, MCIs, MCIp, and AD). Furthermore, field strength-related differences in tissue concentrations and volume measures will be small compared to those related to atrophy, and so seem unlikely to provide significant improvements to classification accuracy. Indeed, we did not observe any unusually impressive results suggestive of such improvements. Finally, we again note that our classification experiments do not need to precisely match the field to be informative.

### 5.4 Population shift

We note that selection bias is distinct from the problem of population shift (or drift) (Hand et al., 2006), which occurs when the feature representations of the subjects to be classified in the real application of a pipeline have a different distribution to that of those used in the validation sample. This is also likely to be a great challenge in the clinical translation of AD classification methods, as they must generalise to scanner types and patient populations that may be unlike those in research settings (Abdulkadir et al., 2011), but it is separate from the issues addressed here.

## 6 Conclusion

We have demonstrated that selection bias can plausibly account for a considerable fraction of the progress associated with pipeline optimisation in AD classification. We remind classification researchers to be aware of the potential for bias in their own work. Specifically, we encourage researchers to use low variance (low K, high repetition) cross validation strategies, and caution against pipeline optimisation or comparison before sufficiently large samples are available, as this will quickly produce misleading performance results while providing little real improvement. We also urge caution in the interpretation of published results; for MCI prognosis in particular, our results suggest that typical sample sizes provide great potential for bias. Before any published pipeline is considered for use in clinical decision making, it is imperative that the potential for selection bias be taken into account. Finally, our results highlight the importance of classification challenges, which offer a rare opportunity for the non-selective reporting of performance results.

While this study focused on AD classification, selection bias is a potential problem for many similar applications of supervised learning in medical imaging. Our results may be useful to researchers in other applications, as the determining factors and possible solutions are likely to be the same.

## Figures and Tables

**Fig. B.12:**
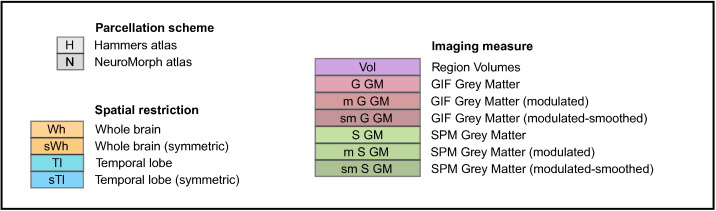
Key to accuracy Fig. B.13, Fig. B.14. Each feature set is described by one label from each group.

**Fig. B.13:**
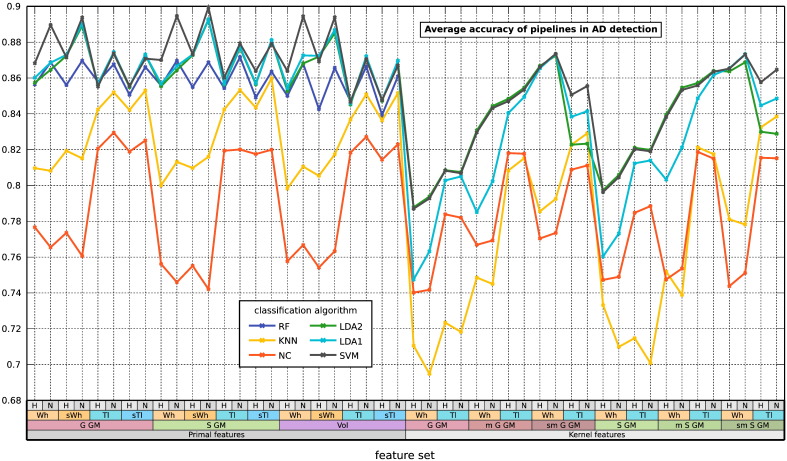
Average accuracies of AD diagnosis pipelines measured using 4×6cv and 300 subjects. See key in Fig. B.12.

**Fig. B.14:**
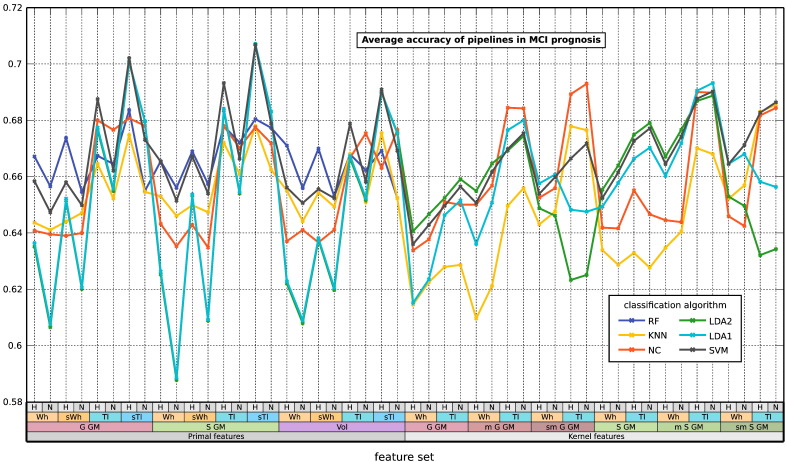
Average accuracies of MCI prognosis pipelines measured using 4×6cv with 160 subjects. See key in Fig. B.12.

## Appendix A Imaging and clinical data

Imaging and clinical data were obtained from the ADNI database. ADNI was launched in 2003 by the National Institute on Ageing, the National Institute of Biomedical Imaging and Bioengineering, the Food and Drug Administration, private pharmaceutical companies and non-profit organisations, as a $60 million, 5-year public/private partnership. The primary goal of ADNI has been to test whether serial MRI, PET, other biological markers, and clinical and neuropsychological assessment can be combined to measure the progression of mild MCI and early AD. Determination of sensitive and specific markers of very early AD progression is intended to aid researchers and clinicians to develop new treatments and monitor their effectiveness, as well as lessen the time and cost of clinical trials. The Principal Investigator of this initiative is Michael W. Weiner, MD, VA Medical Center and University of California at San Francisco. ADNI is the result of efforts of many coinvestigators from a broad range of academic institutions and private corporations, and subjects have been recruited from over 50 sites across the U.S. and Canada. Up-to-date information on the initiative is available at www.adni-info.com.

All subjects were designated as healthy control (HC), AD or MCI at the time of the baseline scan, and were subsequently reassessed at timepoints during follow-up. Inclusion criteria for HC subjects are MMSE scores between 24 and 30, a CDR of 0, non-depressed and non-demented. Ages of the HC subjects were roughly matched to those of the AD and MCI subjects. For MCI subjects, the criteria are an MMSE score between 24 and 30, a memory complaint, objective memory loss measured by education adjusted scores on Wechsler Memory Scale Logical Memory II, a CDR of 0.5, absence of significant levels of impairment in other cognitive domains, essentially preserved activities of daily living, and an absence of dementia. AD subjects were identified by an MMSE score between 20 and 26, CDR of 0.5 or 1.0, and the NINCDS/ADRDA criteria for probable AD.

## Appendix B Average performance of included pipelines

We present the average accuracy of the pipelines in the largest of the samples considered in the resampling design in Fig. B.13, Fig. B.14. A key to understanding these is presented in Fig. B.12. Note that the 3:2 ratio of negative (HC/MCIs) to positive (AD/MCIp) classes should give a “null” accuracy of 60% for both tasks. Our design allows us to produce an unbiased variance estimate for the performance as estimated in a single CV experiment (Mendelson et al., 2014). This in turn allows us to produce a very conservative (upwardly biased) estimate for the variance of our final performance estimate (by falsely assuming the average of all 2000 CV experiment pairs has the same variance as the average of a single pair). This conservative estimate gives standard deviations in the range 1 to 3.5% for all accuracy estimates displayed in this section. For the AD detection task (Fig. B.13) most of these are under 2%; for MCI prognosis (Fig. B.14), most of these are above 2%.

Though we present them primarily to demonstrate the plausibility of our methods, we can also make some comments on the accuracies observed. These span a wide range in both tasks, with those in AD detection spanning the range 70–90%, and those in MCI prognosis spanning the range 60–70%. This is towards the lower end of the spectrum of published results (Arbabshirani et al., 2016, Cuingnet et al., 2011, Falahati et al., 2014, Weiner et al., 2013).

A loose hierarchy of classifier algorithms is evident in the AD detection task, with SVM, LDA and RF consistently performing better than Centroid and KNN with a given feature set. This is not so much the case for MCI prognosis, where the ranking of algorithms is highly dependent on the feature set. This may be due to the more difficult nature of the task confounding the fitting of more complex models. In both cases, clear repeating patterns are visible, with GIF GM, SPM GM and region volumes producing similar results with the same regional restrictions.

## Footnotes

2 Note that Nowotny uses ‘overfitting’ in a colloquial sense to indicate the mere presence of selection bias.

3 https://www.kaggle.com/

4 http://cmictig.cs.ucl.ac.uk/wiki/index.php/NiftyReg

5 http://www.fil.ion.ucl.ac.uk/spm/doc/

6 http://brain-development.org/brain-atlases/

7 https://masi.vuse.vanderbilt.edu/workshop2012/index.php/Main_Page

8 The in-sample performance estimates are themselves used to determine the ranks. If the out-of-sample performance estimates did not also increase with rank, this would mean that poor performance in a CV experiment was indicative of good performance in future experiment repetitions.

9 https://competitions.codalab.org/competitions/1471

## References

[bb0005] Abdulkadir A., Mortamet B., Vemuri P., Jack C.R., Krueger G., Klöppel S., Initiative A.D.N. (2011). Effects of hardware heterogeneity on the performance of SVM Alzheimer's disease classifier. Neuro*I*mage.

[bb0010] Ambroise C., McLachlan G.J. (2002). Selection bias in gene extraction on the basis of microarray gene-expression data. Proc. Natl. Acad. Sci..

[bb0015] Arbabshirani M.R., Plis S., Sui J., Calhoun V.D. (2016). Single subject prediction of brain disorders in neuroimaging: promises and pitfalls. NeuroImage.

[bb0020] Arlot S., Celisse A. (2010). A survey of cross-validation procedures for model selection. Stat. Surv..

[bb0025] Bengio Y., Grandvalet Y. (2004). No unbiased estimator of the variance of K-fold cross-validation. J. Mach. Learn. Res..

[bb0030] Braak H., Braak E. (1991). Neuropathological stageing of Alzheimer-related changes. Acta Neuropathol..

[bb0035] Breiman L. (2001). Random forests. Mach. Learn..

[bb0040] Bron E.E., Smits M., Van Der Flier W.M., Vrenken H., Barkhof F., Scheltens P., Papma J.M., Steketee R.M., Orellana C.M., Meijboom R. (2015). Standardized evaluation of algorithms for computer-aided diagnosis of dementia based on structural MRI: the CADDementia challenge. Neuroimage.

[bb0045] Button K.S., Ioannidis J.P., Mokrysz C., Nosek B.A., Flint J., Robinson E.S., Munafò M.R. (2013). Power failure: why small sample size undermines the reliability of neuroscience. Nat. Rev. Neurosci..

[bb0050] Cardoso M.J., Leung K., Modat M., Keihaninejad S., Cash D., Barnes J., Fox N.C., Ourselin S., Initiative A.D.N. (2013). STEPS: similarity and truth estimation for propagated segmentations and its application to hippocampal segmentation and brain parcelation. Med. Image Anal..

[bb0055] Cardoso M.J., Wolz R., Modat M., Fox N.C., Rueckert D., Ourselin S. (2012). Geodesic information flows. Proc. of Medical Image Computing and Computer-Assisted Intervention (MICCAI), LNCS.

[bb0060] Cawley G.C., Talbot N.L. (2010). On over-fitting in model selection and subsequent selection bias in performance evaluation. J. Mach. Learn. Res..

[bb0065] Challis E., Hurley P., Serra L., Bozzali M., Oliver S., Cercignani M. (2015). Gaussian process classification of Alzheimer's disease and mild cognitive impairment from resting-state fMRI. Neuro*I*mage.

[bb0070] Chang C.C., Lin C.J. (2011). LIBSVM: a library for support vector machines. ACM Trans. Intell. Syst. Technol..

[bb0075] Chu C., Hsu A.L., Chou K.H., Bandettini P., Lin C. (2012). Does feature selection improve classification accuracy? Impact of sample size and feature selection on classification using anatomical magnetic resonance images. Neuroimage.

[bb0080] Coupé P., Eskildsen S.F., Manjón J.V., Fonov V.S., Collins D.L. (2012). Simultaneous segmentation and grading of anatomical structures for patient's classification application to Alzheimer's disease. Neuro*I*mage.

[bb0085] Cuingnet R., Gerardin E., Tessieras J., Auzias G., Lehéricy S., Habert M.O., Chupin M., Benali H., Colliot O., Initiative A.D.N. (2011). Automatic classification of patients with Alzheimer's disease from structural MRI: a comparison of ten methods using the ADNI database. Neuro*I*mage.

[bb0090] Dai Z., Yan C., Wang Z., Wang J., Xia M., Li K., He Y. (2012). Discriminative analysis of early Alzheimer's disease using multi-modal imaging and multi-level characterization with multi-classifier (M3). Neuro*I*mage.

[bb0095] Dukart J., Mueller K., Barthel H., Villringer A., Sabri O., Schroeter M.L., Initiative A.D.N. (2013). Meta-analysis based SVM classification enables accurate detection of Alzheimer's disease across different clinical centers using FDG-PET and MRI. Psychiatry Res. Neuroimaging.

[bb0100] Easterbrook P., Gopalan R., Berlin J., Matthews D. (1991). Publication bias in clinical research. Lancet.

[bb0105] Falahati F., Westman E., Simmons A. (2014). Multivariate data analysis and machine learning in Alzheimer's disease with a focus on structural magnetic resonance imaging. J. Alzheimers Dis..

[bb0110] Frisoni G.B., Fox N.C., Jack C.R., Scheltens P., Thompson P.M. (2010). The clinical use of structural MRI in Alzheimer disease. Nat. Rev. Neurol..

[bb0115] Gousias I.S., Rueckert D., Heckemann R.A., Dyet L.E., Boardman J.P., Edwards A.D., Hammers A. (2008). Automatic segmentation of brain MRIs of 2-year-olds into 83 regions of interest. Neuro*I*mage.

[bb0120] Gray K.R., Aljabar P., Heckemann R.A., Hammers A., Myers R., Rueckert D. (2013). Random forest-based similarity measures for multi-modal classification of Alzheimer's disease. Neuro*I*mage.

[bb0125] Hammers A., Allom R., Koepp M.J., Free S.L., Myers R., Lemieux L., Mitchell T.N., Brooks D.J., Duncan J.S. (2003). Three-dimensional maximum probability atlas of the human brain, with particular reference to the temporal lobe. Hum. Brain Mapp..

[bb0130] Hand D.J. (2006). Classifier technology and the illusion of progress. Stat. Sci..

[bb0135] Kambeitz J., Kambeitz-Ilankovic L., Leucht S., Wood S., Davatzikos C., Malchow B., Falkai P., Koutsouleris N. (2015). Detecting neuroimaging biomarkers for schizophrenia: a meta-analysis of multivariate pattern recognition studies. Neuropsychopharmacology.

[bb0140] Keihaninejad S., Heckemann R.A., Gousias I.S., Hajnal J.V., Duncan J.S., Aljabar P., Rueckert D., Hammers A. (2012). Classification and lateralization of temporal lobe epilepsies with and without hippocampal atrophy based on whole-brain automatic MRI segmentation. PLoS ONE.

[bb0145] Klöppel S., Abdulkadir A., Jr., C.R.J., Koutsouleris N., Mourao Miranda J., Vemuri P. (2012). Diagnostic neuroimaging across diseases. NeuroImage.

[bb0150] Klöppel S., Stonnington C.M., Chu C., Draganski B., Scahill R.I., Rohrer J.D., Fox N.C., Jack C.R., Ashburner J., Frackowiak R.S. (2008). Automatic classification of MR scans in Alzheimer's disease. Brain.

[bb0155] Kriegeskorte N., Simmons W.K., Bellgowan P.S., Baker C.I. (2009). Circular analysis in systems neuroscience: the dangers of double dipping. Nat. Neurosci..

[bb0160] Ledoit O., Wolf M. (2004). A well-conditioned estimator for large-dimensional covariance matrices. J. Multivar. Anal..

[bb0165] Lemm S., Blankertz B., Dickhaus T., Müller K.R. (2011). Introduction to machine learning for brain imaging. NeuroImage.

[bb0170] Mendelson A., Zuluaga M., Thurfjell L., Hutton B., Ourselin S. (2014). The empirical variance estimator for computer aided diagnosis: lessons for algorithm validation. Medical image computing and computer-assisted intervention: MICCAI... International Conference on Medical Image Computing and Computer-Assisted Intervention.

[bb0175] Mendelson, A.F. A list of publications describing new supervised learning pipelines to predict clinical variables from neuroimaging data in Alzheimer's disease doi:10.6084/m9.figshare.3435752.

[bb0180] Nowotny, T. Two challenges of correct validation in pattern recognition. Front. Robotics AI 1 (5). 10.3389/frobt.2014.00005. http://www.frontiersin.org/computational_intelligence/10.3389/frobt.2014.00005/abstract.

[bb0185] Orrù G., Pettersson-Yeo W., Marquand A.F., Sartori G., Mechelli A. (2012). Using support vector machine to identify imaging biomarkers of neurological and psychiatric disease: a critical review. Neurosci. Biobehav. Rev..

[bb0190] Ota K., Oishi N., Ito K., Fukuyama H., S.J.S. Group, Initiative A.D.N. (2015). Effects of imaging modalities, brain atlases and feature selection on prediction of Alzheimer's disease. J. Neurosci. Methods.

[bb0195] Padilla P., López M., Górriz J.M., Ramirez J., Salas-Gonzalez D., Álvarez I. (2012). NMF-SVM based CAD tool applied to functional brain images for the diagnosis of Alzheimer's disease. IEEE Trans. Med. Imaging.

[bb0200] Rao R.B., Fung G., Rosales R. (2008). On the dangers of cross-validation. An experimental evaluation. SIAM International Conference on Data Mining, Society for Industrial and Applied Mathematics.

[bb0205] Shi F., Liu B., Zhou Y., Yu C., Jiang T. (2009). Hippocampal volume and asymmetry in mild cognitive impairment and Alzheimer's disease: meta-analyses of MRI studies. Hippocampus.

[bb0210] Sterne J.A., Egger M. (2001). Funnel plots for detecting bias in meta-analysis: guidelines on choice of axis. J. Clin. Epidemiol..

[bb0215] Weiner M.W., Veitch D.P., Aisen P.S., Beckett L.A., Cairns N.J., Green R.C., Harvey D., Jack C.R., Jagust W., Liu E. (2013). The Alzheimer's disease neuroimaging initiative: a review of papers published since its inception. Alzheimers Dement..

